# Integrated Analysis of the miRNAome and Transcriptome Reveals miRNA–mRNA Regulatory Networks in *Catharanthus roseus* Through *Cuscuta campestris*-Mediated Infection With “*Candidatus* Liberibacter asiaticus”

**DOI:** 10.3389/fmicb.2022.799819

**Published:** 2022-03-03

**Authors:** Chunhua Zeng, Haodi Wu, Mengji Cao, Changyong Zhou, Xuefeng Wang, Shimin Fu

**Affiliations:** National Citrus Engineering Research Center, Citrus Research Institute, Southwest University/Chinese Academy of Agricultural Sciences, Chongqing, China

**Keywords:** huanglongbing, periwinkle, miRNA–mRNA regulatory network, “*Candidatus* Liberibacter asiaticus” (CLas), defense response

## Abstract

Citrus Huanglongbing (HLB) is the most devastating disease of citrus caused by the Gram-negative phloem-limited bacterium “*Candidatus* Liberibacter asiaticus” (CLas). It can be transmitted by the Asian citrus psyllid “*Diaphorina citri*,” by grafting, and by the holoparasitic dodder. In this study, the non-natural host periwinkle (*Catharanthus roseus*) was infected via dodder (*Cuscuta campestris*) from CLas-infected citrus plants, and the asymptomatic leaves (AS) were subjected to transcriptomic and small-RNA profiling. The results were analyzed together with a transcriptome dataset from the NCBI repository that included leaves for which symptoms had just occurred (S) and yellowing leaves (Y). There were 3,675 differentially expressed genes (DEGs) identified in AS, and 6,390 more DEGs in S and further 2109 DEGs in Y. These DEGs were commonly enriched in photosystem, chloroplast, membrane, oxidation-reduction process, metal/zinc ion binding on GO. A total of 14,974 DEGs and 336 DE miRNAs (30 conserved and 301 novel) were identified. Through weighted gene co-expression network and nested network analyses, two critical nested miRNA–mRNA regulatory networks were identified with four conserved miRNAs. The primary miR164-NAC1 network is potentially involved in plant defense responses against CLas from the early infection stage to symptom development. The secondary network revealed the regulation of secondary metabolism and nutrient homeostasis through miR828-MYB94/miR1134-HSF4 and miR827-ATG8 regulatory networks, respectively. The findings discovered new potential mechanisms in periwinkle–CLas interactions, and its confirmation can be done in citrus–CLas system later on. The advantages of periwinkle plants in facilitating the quick establishment and greater multiplication of CLas, and shortening latency for disease symptom development make it a great surrogate for further studies, which could expedite our understanding of CLas pathogenesis.

## Introduction

Citrus Huanglongbing (HLB) is one of the most destructive globally distributed citrus diseases and is responsible for tremendous economic losses to citrus industries worldwide (Wang, 2019). HLB is associated with three species of phloem-limited Gram-negative α*-*Proteobacteria, namely *Candidatus* Liberibacter asiaticus (CLas), *Candidatus* L. americanus (CLam), and *Candidatus* L. africanus (CLaf) (Bové, 2006). CLas is the most widely distributed and is vectored by the Asian citrus psyllid, *Diaphorina citri*. HLB affects almost all commercial citrus and citrus relatives within the family Rutaceae, including the ornamental jasmine (*Murraya paniculata*) (Zhou et al., 2007). Disease symptoms include blotchy mottle, yellow shoots, zinc deficiency, stunting, and twig dieback of citrus plants, as well as the dramatic decrease of fibrous root mass (Johnson et al., 2014; Wu et al., 2018).

Dodder is a holoparasitic plant with a simplified morphology consisting of a stem and haustorium (Sun et al., 2018). Its importance was emphasized with its application as an experimental tool to widen the host range of viruses (Hosford, Jr., 1967) and phytoplasmas (Kamińska and Korbin, 1999) so that they could be studied in a more amenable host. In addition to natural transmission by *D. citri*, CLas also can be transmitted by graft inoculation within the same host genus and via at least three species of dodder (*Cuscuta campestris, Cuscuta pentagona*, and *Cuscuta indecora*) from citrus to non-natural host plants, including periwinkle (*Catharanthus roseus*) (Garnier and Bové, 1983; Hartung et al., 2010), tomato (*Solanum lycopersicum*) (Duan et al., 2008), and tobacco (*Nicotiana xanthi* and *Nicotiana benthamiana*) (Francischini et al., 2007; Pitino et al., 2018). All three non-natural host plants develop HLB-like symptoms, particularly periwinkle, in which CLas colonizes at a higher concentration than in the other two. Therefore, periwinkle has been used as a surrogate for studying phylogenetic and taxonomic characteristics (Teixeira et al., 2008), distribution patterns (Ya et al., 2018), new detection method evaluation (Ding et al., 2017; Gottwald et al., 2020), genomic DNA enrichment and pathogenic effector screening of CLas (Zhang et al., 2011b; Jain et al., 2015), and therapeutic compound/molecule screening for HLB control (Zhang et al., 2010; Zhang et al., 2011a).

Although CLas has been studied for a century, no curable way is available so far. CLas has not been cultured *in vitro*, therefore Koch’s postulates have not been completed and little is known about its pathogenesis through traditional molecular and genetic analyses (Wang et al., 2017). With the application of high-throughput sequencing (HTS) approaches, a large number of transcriptomic studies and a few small-RNA studies have been conducted on different citrus varieties in response to CLas. However, there has been only one transcriptomic profiling study where periwinkle was infected by CLas (Liu et al., 2019), and no combined transcriptomic and small-RNA studies have been reported. Therefore, we simultaneously analyzed the transcriptome and miRNAome of CLas-affected periwinkle in this study. The characterized miRNA-mediated networks could provide valuable information for better understanding the molecular regulatory mechanism of periwinkle in response to CLas infection.

## Materials and Methods

### Plant Materials and Treatments

#### *Candidatus* Liberibacter asiaticus Inoculation on Periwinkle Through Dodder Bridge Transmission

The CLas strain was originally collected from a virus-free HLB-infected “Guanximiyou” pomelo plant from Ganzhou, Jiangxi Province, China. The CLas-infected budwoods were grafted onto two-year-old sweet orange seedlings (*Citrus sinensis*), and its presence was verified by PCR with the primer pair OI1/OI2c (Jagoueix et al., 1996).

**FIGURE 1:**
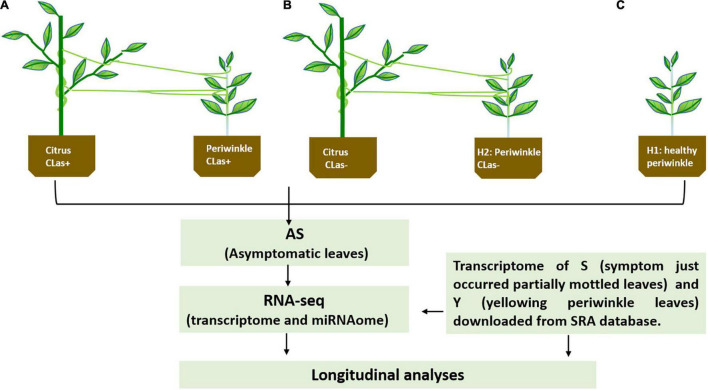
**(A,B)** Schematics for the experimental setup and downstream analyses. CLas+ and CLas- represent *Candidatus Liberibacter* asiaticus positive and negative, respectively, by PCR test; H1, healthy periwinkle not parasitic with healthy dodder as control; H2, healthy periwinkle parasitic with healthy dodder as control.

Dodder (*C. campestris*) was placed on two-year-old healthy periwinkle plants (*C. roseus*) after germination from the seeds to obtain more tendrils, and then some of the tendrils were transferred to another healthy periwinkle. After approximately 1 week, the dodder successfully formed haustoria and parasitized the periwinkle, and its tendrils were manually connected to CLas-infected citrus plants (Li et al., 2021). One week after connection, the dodder bridge was completely established (Figure 1A). Healthy periwinkle plants (H2) were also connected to healthy citrus plants through healthy dodder (Figure 1B), and healthy periwinkle plants (H1) not parasitized with dodder (Figure 1C) were set as controls. As dodder is a stressor for host plants and is capable of mediating the trafficking of mobile mRNA, small-RNAs, and proteins between hosts and parasitic plants, it likely plays important roles in coordinating plant growth, development, and adaptation to stressors (Liu et al., 2020). All plants were maintained in the greenhouse at the Citrus Research Institute in Chongqing, China. Temperatures were maintained between 25 and 30°C, and supplemental lighting was provided. The potting mix contained turf soil, sand, and rice husks (1:1:1). Chemical fertilizers with a macronutrient content of 12:12:12 (NPK) were used twice per month, and rapeseed dregs were added to supply microelements.

#### *Candidatus* Liberibacter asiaticus Detection and RNA Collection

The presence of CLas on dodder (about 40 days of dodder bridge assembly) and periwinkles (approximately 3 months of dodder bridge assembly) was verified by PCR with the primer pair OI1/OI2c (Jagoueix et al., 1996) (Supplementary Figure 1). Three newly flushed, fully expanded, and asymptomatic periwinkle leaves (AS) from infected plants (*n* = 3, Figure 1A), non-infected plants parasitized with dodder (*n* = 3, Figure 1B), and non-infected plants not parasitized with dodder (*n* = 3, Figure 1C) were sampled. Total RNA was extracted with TRIzol reagent (Invitrogen, United States) according to standard procedure, and residual DNA was removed with DNAseI (Takara, Shiga, Japan). The quantity and quality of the total RNA were determined with a NanoDrop ND-1000 and Agilent 2100 Bioanalyzer (Thermo Fisher Scientific, MA, United States), respectively. The nine RNA extracts, which included three biological replicates for each treatment, were sent to BGI (Shenzhen, China) for both transcriptome and small-RNA library construction and sequencing.

#### RNA Library Construction and Sequencing

Five micrograms of total RNA were used for transcriptome library preparation with a TruSeq RNA sample preparation kit (Illumina, San Diego, CA, United States) following the manufacturer’s protocol. The small-RNA libraries were constructed with a TruSeq Small-RNA Library Prep Kit according to the manufacturer’s instructions (Illumina, San Diego, CA, United States). The obtained cDNA libraries were directly sequenced on a BGISEQ-500 platform (BGI-Shenzhen, China) with paired-end 150 bp and single-end 50 bp reads for mRNA and small-RNA, respectively.

#### Bioinformatics Analyses

For transcriptomic profiling, the raw reads were adapter-trimmed and quality-filtered with Trimmomatic (Bolger et al., 2014). The clean reads were mapped to the reference genome of *C. roseus* (She et al., 2019) using CLC Genomics Workbench 20.0.4 (Qiagen, Hilden, Germany) with the default parameters. The expression of transcripts was qualified with the reads per kilobase per million mapped reads (RPKM) method (Mortazavi et al., 2008), and differentially expressed genes (DEGs) were filtered by | log2(FC)| ≥ 0, *P*-value < 0.05, and false discovery rate (FDR) < 0.1. The functions of the DEGs were enriched in Gene ontology (GO) database, and mapping to *C. roseus* with MapMan 4.0 and the mapping files of *C. roseus* were annotated with the PlaBi database by Mercator4 V3.0 (Schwacke et al., 2019). The transcriptomic dataset with nine periwinkle samples infected with CLas by grafting was downloaded from the NCBI database and processed as above (Liu et al., 2019). It contained three groups, with three replicates for each group, and included early symptoms on partially mottled leaves (S), yellow periwinkle leaves (Y), and healthy periwinkle leaves (H).

For the small-RNA datasets, reads shorter than 18 nt or longer than 32 nt were removed using Trimmomatic (Bolger et al., 2014) and then filtered for rRNA, tRNA, snoRNA, snRNA, repeat sequences, and other ncRNAs using Pfam v.14.0 with the default parameters (Kalvari et al., 2021). The remaining reads were mapped to known miRNAs from the miRBase database to identify conserved miRNAs (Kozomara and Griffiths-Jones, 2014) and further analyzed to identify novel miRNAs using miR-PREFer v. 0.24 with the default parameters (Lei and Sun, 2014). The identified conserved and novel miRNAs were compared against *C. roseus* mRNA transcript coding sequences with psRNATarget v.2.0 (Dai et al., 2018). The differential expression of miRNAs was analyzed using DEseq2 with a cutoff value of *P* < 0.05 (Love et al., 2014). The annotated mRNA targets were identified using Blast2Go (Götz et al., 2008).

#### Nested Network Analysis of Differentially Expressed Genes and miRNAs

Genes with an expression value (RPKM) < 1 from all expressed genes were filtered out, and the remaining genes were used to infer a weighted gene co-expression network analysis WGCNA (signed network) using an online tool^1^ with the default parameters. An appropriate soft power = 1 was automatically selected to make the scale-free topology index greater than 0.8. The WGCNA algorithm was used to construct gene modules and evaluate the connectivity of genes in a module. In our study, CLas infection and dodder parasitism were considered the two traits among different sample groups, and then the module eigengene (ME) value was calculated and used to test the association with different traits. After the MEs with the highest relationship to the CLas infection trait were determined, | geneModuleMembership| > 0.8 and | geneTraitCorrelation| > 0.2 were used as the cutoff values for gene member selection in each module. Overlapping components of the DEGs and miRNA-target in the CLas infection groups versus the control group were selected from MEs to construct the nested network using Cytoscape v3.8.2 (Kohl et al., 2011).

#### Validation of Differentially Expressed Genes, miRNAs, and Their Target Genes in *Catharanthus roseus*

For expression analysis, real-time quantitative PCR (RT-qPCR) (Fu et al., 2016) and stem-loop RT-qPCR (Kramer, 2011) were applied for DEGs and miRNA, respectively, with the same set of samples used for RNA-seq. The expression of both the DEGs and miRNAs was normalized with the U6 small nuclear ribonucleic acid (snRNA) gene. The primers used in this study are listed in Supplementary Table 1. The first strand was synthesized using the PrimeScript RT reagent kit with a gDNA Eraser (Takara, Shiga, Japan) for both mRNA and miRNA according to the manufacturer’s protocol. The RT-qPCR was conducted on a qTower3G platform (Analytikjena, Jena, Germany) with GoTaq qPCR Master Mix (Promega, China) for mRNA and miRNA. All experiments were performed with three biological replicates and three technical replicates. Specificity of primers was determined with melting curves. The 2^–△△Ct^ method was applied for relative quantification, with the U6 small nuclear ribonucleic acid (snRNA) gene as an internal reference (Livak and Schmittgen, 2001). Expression fold change was further used to analyze the differences between the treatment group and control group.

## Results

### Overview of Transcriptome and miRNAome Profiling

For transcriptomic profiling, 40–47 million clean reads were obtained from each plant with/without CLas infection, and approximately 56–60% (average of three biological replicates) of reads were successfully mapped to the *C. roseus* genome (Supplementary Table 2). For the small-RNA datasets, 20–26 million clean reads were obtained, and approximately 65–82% (average of three biological replicates) of reads were successfully aligned to the *C. roseus* genome (Supplementary Table 2).

### Identification of Differentially Expressed Genes and Differentially Expressed miRNAs and Functional Analyses

**FIGURE 2:**
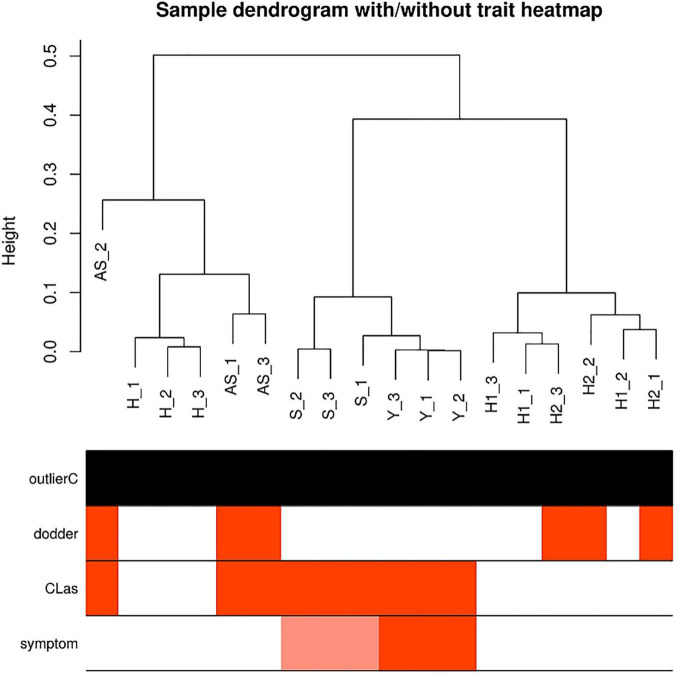
Sample correlations evaluated with dendrogram. Red bars in each row indicate samples in relation to factors, including dodder, CLas and symptom. None is detected as outlier samples. H1, healthy periwinkle not parasitic with healthy dodder as control; H2, healthy periwinkle parasitic with healthy dodder as control; AS, asymptomatic periwinkle infected with CLas by dodder-mediated transmission. H, healthy periwinkle leaves; S, symptom just occurred partially mottled leaves; Y, yellow periwinkle leaves.

Sample correlations were evaluated for eighteen samples (Figure 2), and the DEGs for four comparisons were determined (Table 1). Healthy periwinkle H1 and H2 were clustered together, and the number of DEGs was approximately the same in the asymptomatic samples AS/H1 and AS/H2, indicating that dodder parasitism makes no significant effect on periwinkle in the process of CLas transmission. Although symptomatic samples S were slightly varied from Y, both were still closely clustered and away from asymptomatic samples AS. The number of DEGs was doubled in the S/H and Y/H compared to AS/H1(AS/H2). The results indicated that more genes and corresponding pathways were perturbed as symptom development.

**TABLE 1:** Number of regulated genes and miRNAs in periwinkle infected with “*Candidatus* Liberibacter asiaticus.”

Sample	Number of DEGs					Number of DE miRNA							DEG/total targets
	Up	Down	Co-	S^+^	Y^+^	Up	Down	Total	Co-	Known	Novel	Total	
AS/H1	2,696	2,301				88	66	154					
AS/H2	2,558	2,577	3,657			58	209	267	85	30	301	336	
S/H	3,791	6,324		6,390									
Y/H	4,091	7,017	4,895		2,109								720/2144

*DEGs, differentially expressed genes; DE miRNA, differentially expressed miRNA; H1, healthy periwinkle not parasitic with healthy dodder as control; H2, healthy periwinkle parasitic with healthy dodder as control; AS, asymptomatic periwinkle infected with CLas by dodder-mediated transmission; H, healthy periwinkle leaves; S, symptom just occurred partially mottled leaves; Y, yellow periwinkle leaves; S^+^, more DEGs identified in S samples; Y^+^, more DEGs identified in Y samples; co-, commonly regulated DEGs or DE miRNA.*

**FIGURE 3:**
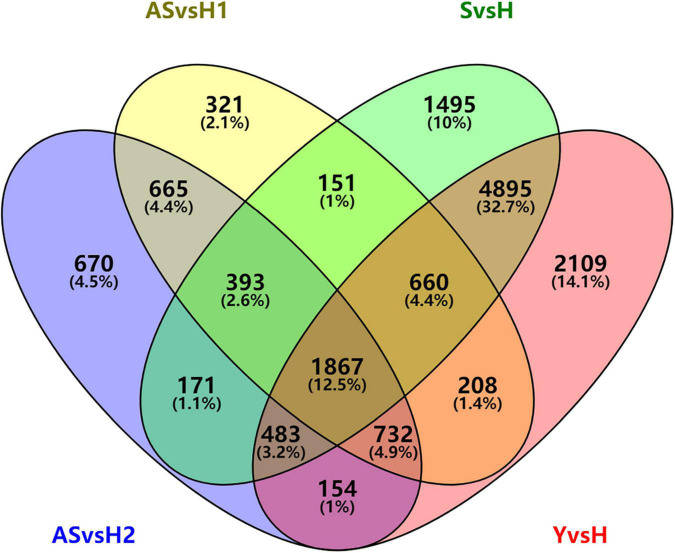
Venn diagram of differentially expressed genes in periwinkle infected with “*Candidatus* Liberibacter asiaticus.”

There were 3,657 DEGs were commonly regulated in AS/H1 and AS/H2. They were enriched in oxidation-reduction process, protein phosphorylation and photosynthesis on the biological process (BP) level, in integral component of membrane, plasma membrane, chloroplast (envelop and thylakoid membrane) on the cellular component (CC) level, in ATP binding, metal ion binding and protein serine/threonine kinase activity on molecular function (MF) level (Supplementary Figure 2). There were 6,390 more DEGs were identified in S/H than in AS/H1(AS/H2). These DEGs were enriched in oxidation-reduction process, protein phosphorylation, and translation on the BP level, in integral component of membrane, nucleus/plasma membrane/cytoplasm, chloroplast/mitochondrion on the CC level, in ATP binding, zinc ion binding, protein serine/threonine kinase activity/nucleotide binding on the MF level (Supplementary Figure 3). As the further development of symptoms, 2,109 more DEGs were identified in Y/H than in S/H and they were enriched in oxidation-reduction process, photosynthesis and related pathways on the BP level, in integral component of membrane, chloroplast/cytoplasm/mitochondrion, and golgi apparatus on the CC level (Supplementary Figure 4). There were 1,867 DEGs that were commonly regulated and 14,974 DEGs that were overall regulated among the four comparisons (Figure 3), and they were also enriched on the three levels of GO (Supplementary Figures 5, 6). These DEGs were also assigned to different pathways in MapMan (Supplementary Table 3). Generally, photosynthesis-, secondary metabolism-, and cell wall organization-related pathways were down-regulated, while cellular respiration, carbohydrate metabolism, RNA biosynthesis, protein modification, protein homeostasis, vesicle trafficking, and external stimuli response were up-regulated in AS (Supplementary Figure 7A). As symptoms developed in S and Y, it appeared that the up-regulation level of the DEGs was reduced compared to AS (Supplementary Figure 7B), particularly the DEGs related to external stimuli response, which changed from up-regulated to down-regulated (Supplementary Table 3).

In total, 336 (30 conserved and 301 novel) differentially expressed (DE) miRNAs (Table 1) were identified that potentially targeted 2144 *C. roseus* mRNAs, in which 720 were differentially expressed in the transcriptome profiling (Table 1). These DEG targets were mostly enriched in RNA biosynthesis, vesicle trafficking, and solute transport, followed by photosynthesis, carbohydrate metabolism, phytohormone action, protein biosynthesis, and protein homeostasis (Supplementary Table 4).

### miRNA–mRNA Network Analyses

Through WGCNA analysis of 18 transcriptomic expression profiles, three modules were determined, and MEbrown was most highly related to CLas infection, followed by MEblue (Supplementary Figure 8A). Hub genes were also determined for each module (Supplementary Figure 8B). By application of threshold cutoff values of | geneModuleMembership| > 0.8 and | geneTraitCorrelation| > 0.2, 187 and 383 DEGs were selected from MEbrown and MEblue, respectively (Supplementary Table 5) and integrated with the DE miRNA and their differentially expressed targets to construct nested networks in Cytoscape v3.8.2.

**FIGURE 4:**
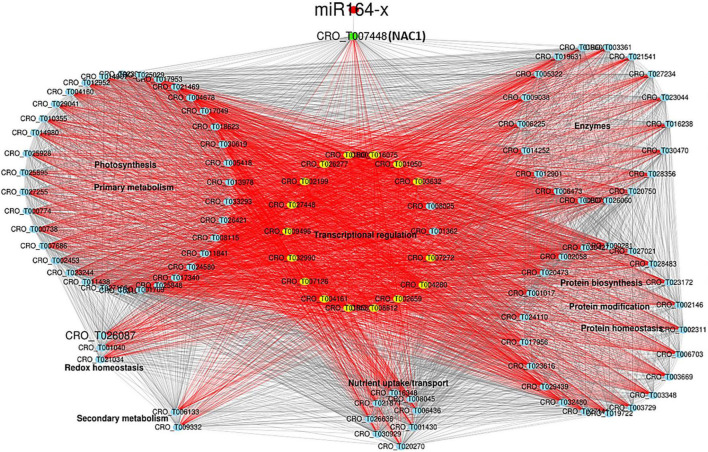
miR164-NAC1 regulatory network highly related to CLas infection. The red nodes are miRNA. The green and yellow nodes are transcription factors (TFs). The blue nodes are genes. Red lines indicate connections to TFs.

The primary network was constructed with DEGs from MEbrown containing six miRNAs (one conserved and five novel) and 154 DEGs (Supplementary Table 6 and Supplementary Figure 9). Within the network, genes in photosynthesis, carbohydrate metabolism, coenzyme metabolism, redox homeostasis, and secondary metabolism were overwhelmingly down-regulated. Three up-regulated novel miRNAs (novel-m1620-5p, novel-m0655-5p, and novel-m0381-5p) targeted a down-regulated chlorophyll *a/b*-binding protein 5 of photosystem I, and a down-regulated novel-m0431-5p targeted a down-regulated chlorophyll a-b binding protein CP29.1. Another induced novel miRNA (novel-m0499-3p) was predicated to target induced nucleoredoxin involved in redox homeostasis. The known miR164-x was repressed and targeted a highly induced transcription factor (TF) NAC1/ANAC022/anac021 (CRO_T007448) (Figure 4 and Supplementary Table 6), which served as the core node connecting a bunch of significantly expressed TFs, including highly expressed HD-ZIP I/II and NAC, and down-expressed bHLH, C2H2, and TCP (Supplementary Table 6), functioning in transcriptional regulation. Genes in protein biosynthesis/homeostasis/modification and nutrient uptake/transport were differentially expressed (Supplementary Table 6).

**FIGURE 5:**
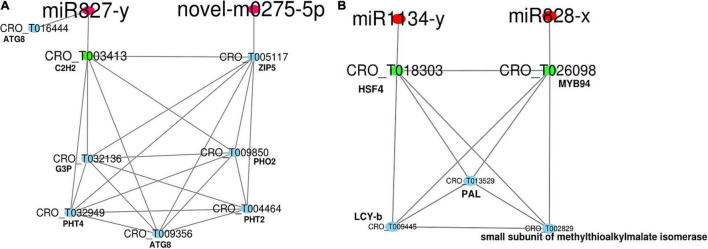
The network of miRNA in the regulation of nutrient and secondary metabolism. The red nodes are miRNAs. The green nodes are transcription factors (TFs). The blue nodes are genes. **(A)** and **(B)** The nutrient and secondary related regulatory networks, respectively. The red nodes are miRNAs. The green nodes are TFs. The blue nodes are genes.

The secondary network was constructed with DEGs from MEblue containing four miRNAs (three conserved and one novel) and 316 DEGs (Supplementary Table 7 and Supplementary Figure 10). Within this network, genes in photosynthesis were generally down-regulated. Many genes in solute transport were differentially expressed. miR827 was Pi deficiency-inducible, and its expression was highly induced. It was predicted to target a down-expressed TF, BALDIBIS/BIB (CRO_T003413) in positive regulation of transcription. A novel miRNA (novel-m0275-5p) was repressed and predicted to target an overexpressed ZIP5 transporter (CRO_T005117). A gene-encoding ubiquitin-conjugating E2 protein (PHO2) in phosphate assimilation was slightly up-regulated, while phosphate transporters (PHT2 and PHT4) were down-regulated (Figure 5A). Redox homeostasis, brassinosteroid and gibberellin phytohormone, and protein modification and homeostasis were disturbed, and genes in ubiquitin-proteasome system, phosphorylation, and S-glutathionylation were particularly up-regulated. Notably, a gene encoding ubiquitin-fold protein (ATG8) involved in autophagy was induced in the symptomatic samples (Supplementary Table 7). The expression of miR828-x was repressed and targeted the down-expressed MYB94 (CRO_T026098), which formed a network with an overexpressed HSF4 (CRO_T018303) and LCY-b (CRO_T009445, lycopene beta cyclase) involved in the terpenoid pathway (Figure 5B). Meanwhile, HSF4 was targeted by the down-expressed miR1134-y. The expression of both effector-triggered immunity co-regulator PAD4 and programmed cell death metacaspase-like regulator MCP2 was induced (Supplementary Table 7).

### Validation of mRNA and miRNA Expression by RT-qPCR

**FIGURE 6:**
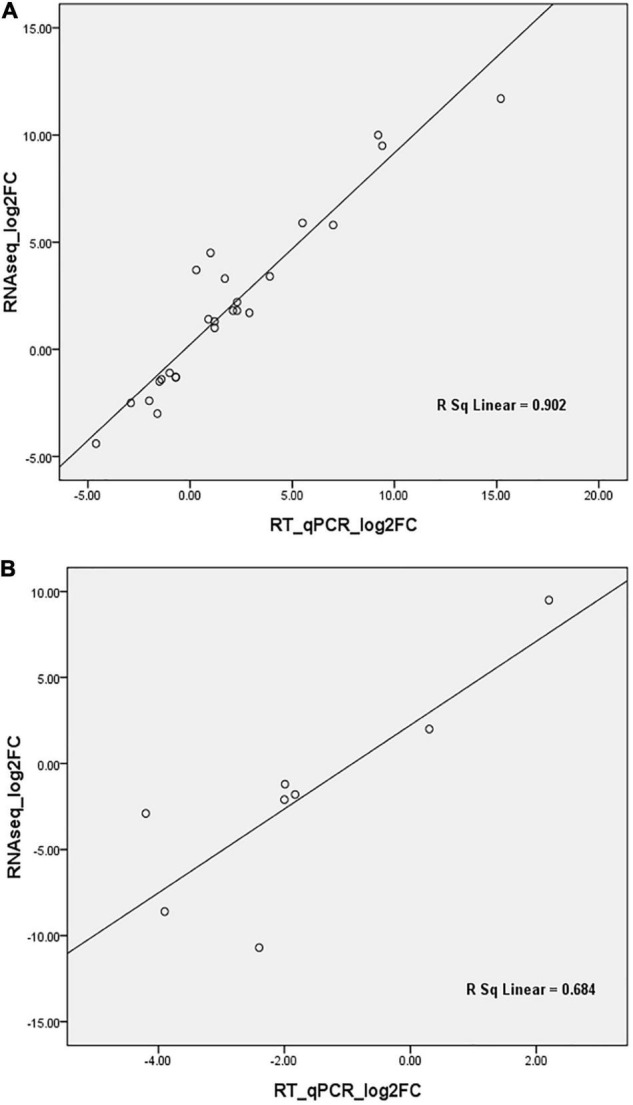
Correlation analyses of differential gene expression by RT-qPCR and RNA-seq. **(A)** verification analyses for differentially expressed genes; **(B)** verification analyses for differentially expressed miRNAs. FC, Fold change.

The expression level of 25 DEGs, 19 of which were the targets of 20 DE miRNAs, was assayed by RT-qPCR. These genes were found to be involved in different biological processes encoding NAC1, ZIP1, ZIP5, light harvesting complex (LHCA6, LHCB4.1), phloem protein (PP2-A13, PP-B15), autophagy-related protein (ATG8), E3 ubiquitin-protein ligase, LecRK-VII.1, MuDR transposase, remorin protein, indole-3-acetic acid inducible, CIPK10, osmotin 34, curculin-like (mannose-binding) lectin, and gibberellin 2-oxidase. The expression of eight DE miRNAs with two conserved miRNAs (miR164-x and miR828-x) was verified by stem-loop RT-qPCR. Spearman’s rho values of 0.9 for mRNA and 0.68 for miRNA (Figure 6) confirmed the reliability of the transcriptomic and small-RNA profiling data. The expression levels of the DEGs and DE miRNAs between the RNA-seq and RT-qPCR are detailed in Supplementary Figure 11.

## Discussion

### The Invasion of *Candidatus* Liberibacter asiaticus and Symptom Development Affect Host Responses Rather Than Transmission Method

Although the 18 transcriptome profiles were collected from two independent experiments with periwinkle infected by CLas either by grafting or dodder bridge, the results showed that all datasets are comparable and dodder does not make significant effect on periwinkle responses during CLas transmission. What does greatly affect host responses is the invasion of CLas and the disease progress from asymptomatic to symptomatic. Prior to symptom occur, photosystem and chloroplast have already been perturbed. With symptom appearance, organelles nucleus, mitochondrion and golgi apparatus were gradually disturbed, but defense response related pathways were not significantly enriched.

### The miR164-NAC1 Network Potentially Regulates Plant Defense Responses to *Candidatus* Liberibacter asiaticus

*ORE1*, an NAC TF, has been demonstrated to positively regulate age-induced cell death in *Arabidopsis* leaves and is negatively regulated by miR164 (Kim et al., 2009). Both miR164 and its target *NAC21/22* were activated, and *NAC21/22* negatively regulates wheat resistance to stripe-rust (Feng et al., 2014). The *Arabidopsis NAC4* promotes pathogen-induced cell death under negative regulation by miR164 (Lee et al., 2017). The miR164a-NAC60 module negatively regulates immunity in rice against the blast fungus *Magnaporthe oryzae* (Wang et al., 2018). The miR164-NAC100 regulatory system modulates cotton plant resistance against *Verticillium dahlia* (Hu et al., 2020). The conserved miR164-NAC regulatory pathway was identified for disease defense in *Populus* during leaf black spot fungus infection (Chen et al., 2021). Plants can sense signals from pathogens during their invasion and activate a complicated and fine-tune network in relation to phytohormones and reactive oxygen species (ROS). NAC TFs have been demonstrated to participate in plant–pathogen interactions as negative and positive regulators by connecting signaling pathways of PCD, plant hormones, and to regulate the resistance against pathogens (Bian et al., 2020). The differential expression of miR164 and NAC1 was identified and verified in AS samples, and further confirmed by (stem-loop) RT-qPCR in another independent set of diseased periwinkle leaves with early blotchy mottle symptom (Supplementary Figure 12). Therefore, the early activation of miR164-NCA1 regulatory network is potentially involved in periwinkle defense responses against CLas, but it couldn’t prevent the multiplication of CLas and symptom development.

### miRNA–mRNA Networks Regulate Secondary Metabolism and Nutrient Homeostasis

miR828 regulates lignin and H_2_O_2_ accumulation in sweet potato upon wounding to participate in defense by targeting *MYB* (Lin et al., 2012). miR828 is also involved in the regulation of trichome and cotton fiber development by targeting *MYB2* in *Arabidopsis* (Guan et al., 2014), as well as anthocyanin and flavonol accumulation by targeting *MYB114* in grape (Tirumalai et al., 2019). As the target of miR828, *MYB* modulate gene expression patterns in the flavonoid biosynthetic pathway (Samad et al., 2020). *MYB94* formed a network with *LCY-b* in terpenoid pathway and *HSF4* targeted by miR1134, which is involved in the terpenoid biosynthetic pathway by miRNA-based regulation (Fan et al., 2015; Samad et al., 2020). The expression of genes in the flavonoid and terpenoid biosynthetic pathways was altered in periwinkle in response to CLas infection (Supplementary Tables 6, 7). Therefore, miR828-MYB94 and miR1134-HSF4 potentially function in the regulation of secondary metabolism in periwinkle against CLas.

miR827 is a phosphate (Pi) starvation-inducible miRNA. The miR827-NAL (nitrogen limitation adaption) and miR827-PHT (phosphate transporter 5) modules were found to maintain cellular Pi homeostasis in *Arabidopsis* and *Oryza sativa* (Lin et al., 2018), respectively. miR399, another Pi deficiency-inducible miRNA, was significantly induced in citrus by CLas infection, and HLB symptom severity was significantly reduced by Pi supplementation (Zhao et al., 2013). The expression of miR399 was induced in periwinkle but was not involved in the identified core network (not shown). Nutrient deficiency, particularly zinc deficiency, is commonly observed in citrus parasitized by CLas. ZIP5, functioning in zinc transportation, was up-regulated in periwinkle, strongly associated with the enrichment of DEGs in zinc ion binding in symptom just occurred samples (Supplementary Figure 3). Zn and Pi transport and homeostasis are recognized as highly co-regulated process and it has been demonstrated that Zn deficiency induced Pi accumulation in *Arabidopsis* (Khan et al., 2014). This result indicates that periwinkle experienced a nutrient imbalance, in particular Pi and Zn deficiency, which partially resulted from the blockage of the phloem system and root decline with the disease development under CLas stress (Johnson et al., 2014).

In addition to the Pi homeostasis regulation, miR827 acts as a negative regulator suppressing basal defense responses in *Arabidopsis*, favoring nematode parasitism through post-transcriptional gene silencing of its ubiquitin E3 ligase target gene (Hewezi et al., 2016). ATG8 is another target of miR827 that is involved in maintaining protein homeostasis through autophagy, which occurs in response to nutrient limitation and bacterial effectors (Üstün et al., 2018). In facilitating *Pseudomonas syringae* pv. tomato (*Pto*) DC3000 infection, effector HrpZ1 oligomerization targets the ATG4b-mediated cleavage of ATG8 to enhance autophagy, while HopF3 targets ATG8 to suppress autophagy (Lal et al., 2020). CLas encodes 166 sec-dependent presumable effectors, and 86 out of 166 were experimentally validated to contain a signal peptide with poorly understood mechanisms (Prasad et al., 2016). One effector was primarily identified to interact with citrus ATG8a (not shown), and the associated molecular mechanism is currently being researched by our laboratory. Thirty-five autophagy-related genes were identified in sweet orange (*C. sinensis*), and CsATG18a and CsATG18b were verified to enhance plant tolerance to abiotic stress (Fu et al., 2020). Effectors play vital roles in the pathogenesis of virulent plant pathogens by targeting host ATG proteins. The activation of the effector-triggered immunity pathway was observed in the current study, but autophagy-related machinery has not been reported either in citrus or in periwinkle by CLas infection, indicating that this could be a promising research focus for future studies.

Currently, the citrus-dodder–periwinkle transmission system has been well-established in our laboratory by optimizing growing conditions. Periwinkle plants can be infected by CLas within two weeks and show symptoms within one month. The early developed symptom on periwinkle resembles the typical blotchy mottle on citrus, then it gradually changes to yellowing (Supplementary Figure 12). Due to the ability of periwinkle to support the quick establishment and the higher colonization level of CLas, and greatly shortening the long latency for disease symptom development on citrus, periwinkle can be used as surrogate for CLas pathogenesis studies in the future.

## Conclusion

Through concurrent analyses of the transcriptome and miRNAome of periwinkle in response to CLas infection, primary and secondary nested miRNA–mRNA networks were identified with four conserved miRNAs. The primary network miR164-NAC1 is potentially involved in plant defense responses against CLas from the early infection stage to symptom development. The secondary network revealed the potential regulation of secondary metabolism and nutrient homeostasis through miR828-MYB94/miR1134-HSF4 and miR827-ATG8 regulatory networks, respectively. By using periwinkle as a surrogate, the identified miRNAs-mRNA regulatory networks provide promising perspectives on host–CLas interactions.

## Data Availability Statement

The RNA-seq datasets supporting the manuscript are available in the SRA database under accession ID PRJNA723735.

## Author Contributions

SF performed the experiments, data analyses, and wrote the manuscript. HW and ChZ prepared the experimental plant settings and collected the data. MC did small RNA data analyses. CyZ and XW conceptualized the idea and revised the manuscript. All authors contributed to the article and approved the submitted version.

## Conflict of Interest

The authors declare that the research was conducted in the absence of any commercial or financial relationships that could be construed as a potential conflict of interest.

## Publisher’s Note

All claims expressed in this article are solely those of the authors and do not necessarily represent those of their affiliated organizations, or those of the publisher, the editors and the reviewers. Any product that may be evaluated in this article, or claim that may be made by its manufacturer, is not guaranteed or endorsed by the publisher.

## Footnotes

1
http://www.ehbio.com/Cloud_Platform/front/#/
