# Supplementary material for: Integrated Analysis of the miRNAome and Transcriptome Reveals miRNA–mRNA Regulatory Networks in Catharanthus roseus Through Cuscuta campestris-Mediated Infection With “Candidatus Liberibacter asiaticus”
Source: Front Microbiol. 2022 Mar 3;13:799819. doi: 10.3389/fmicb.2022.799819 (PMC8928264; doi:10.3389/fmicb.2022.799819)
Supplement: Supplementary file 2 [file Presentation_1.pptx]

## Slide 1
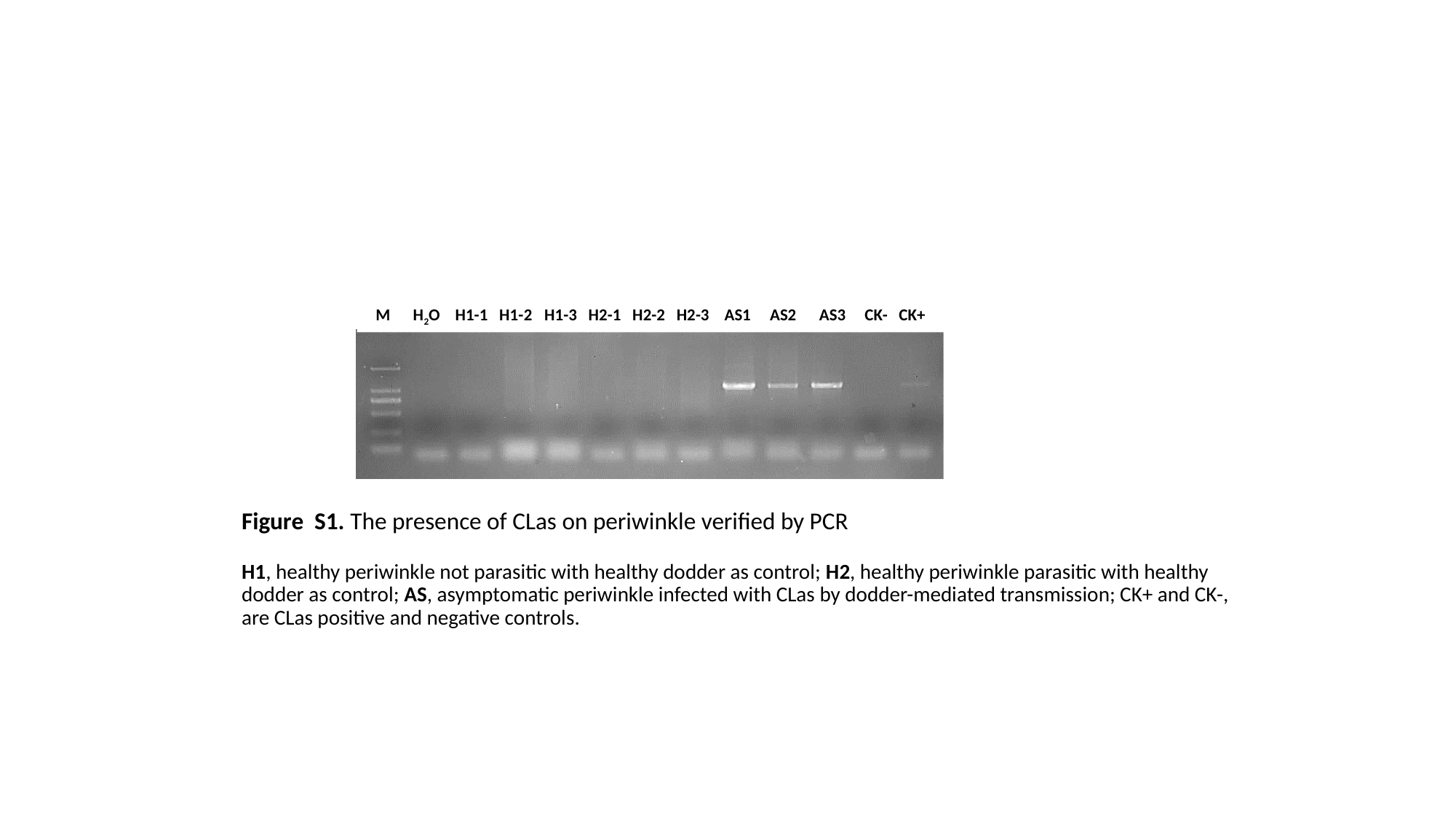

M H2O H1-1 H1-2 H1-3 H2-1 H2-2 H2-3 AS1 AS2 AS3 CK- CK+
# Figure S1. The presence of CLas on periwinkle verified by PCR H1, healthy periwinkle not parasitic with healthy dodder as control; H2, healthy periwinkle parasitic with healthy dodder as control; AS, asymptomatic periwinkle infected with CLas by dodder-mediated transmission; CK+ and CK-, are CLas positive and negative controls.

## Slide 2
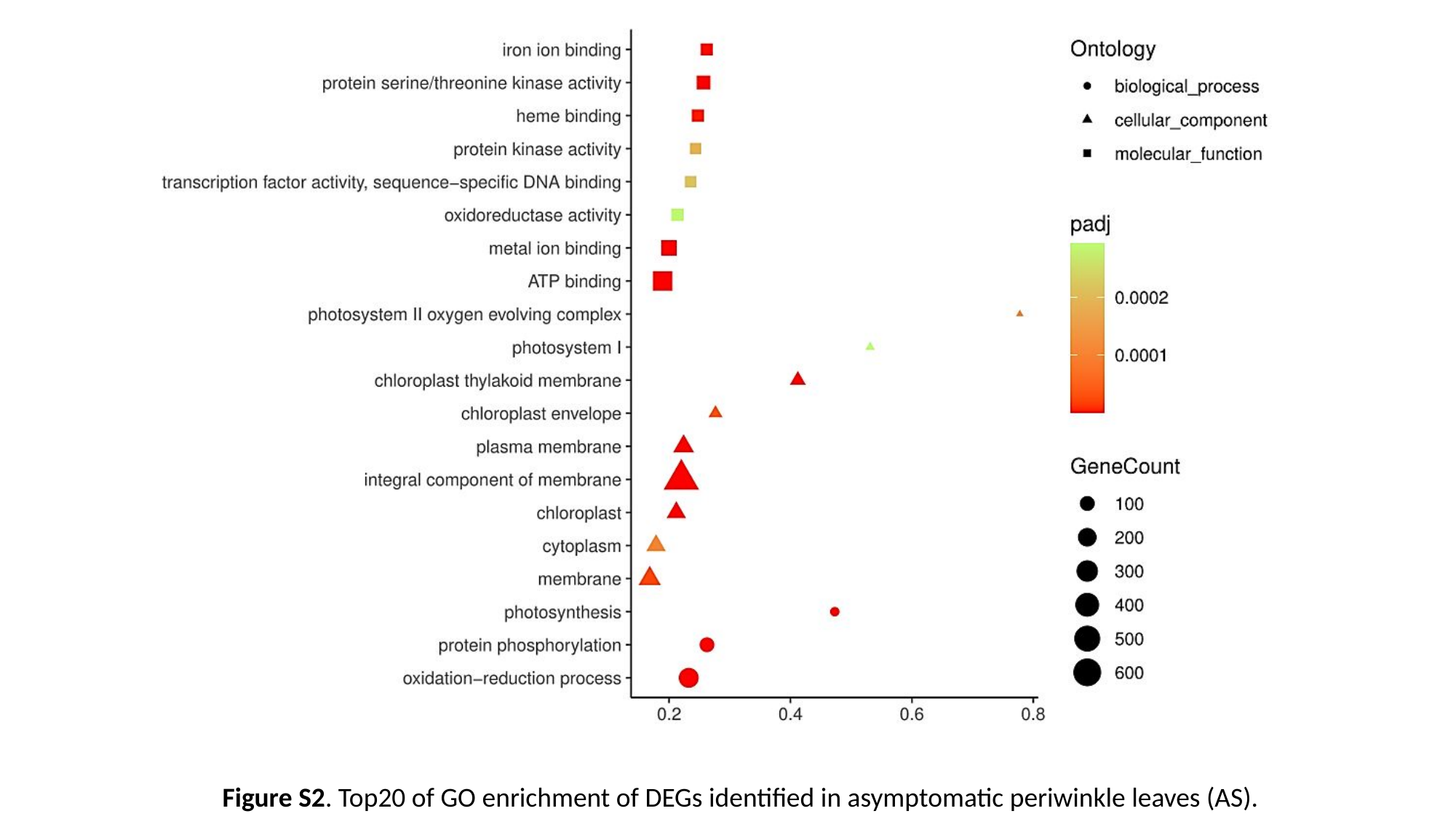

Figure S2. Top20 of GO enrichment of DEGs identified in asymptomatic periwinkle leaves (AS).

## Slide 3
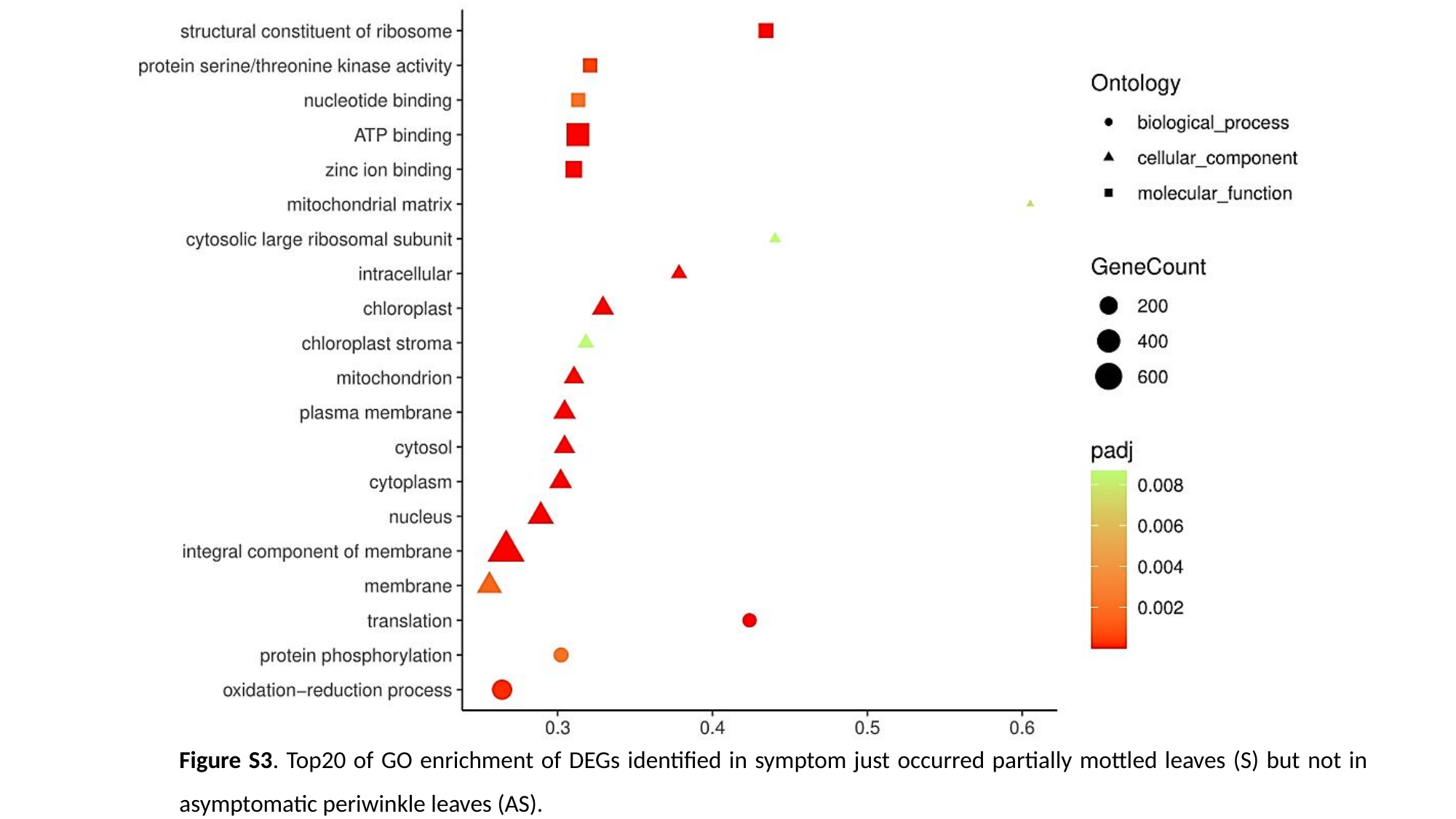

Figure S3. Top20 of GO enrichment of DEGs identified in symptom just occurred partially mottled leaves (S) but not in asymptomatic periwinkle leaves (AS).

## Slide 4
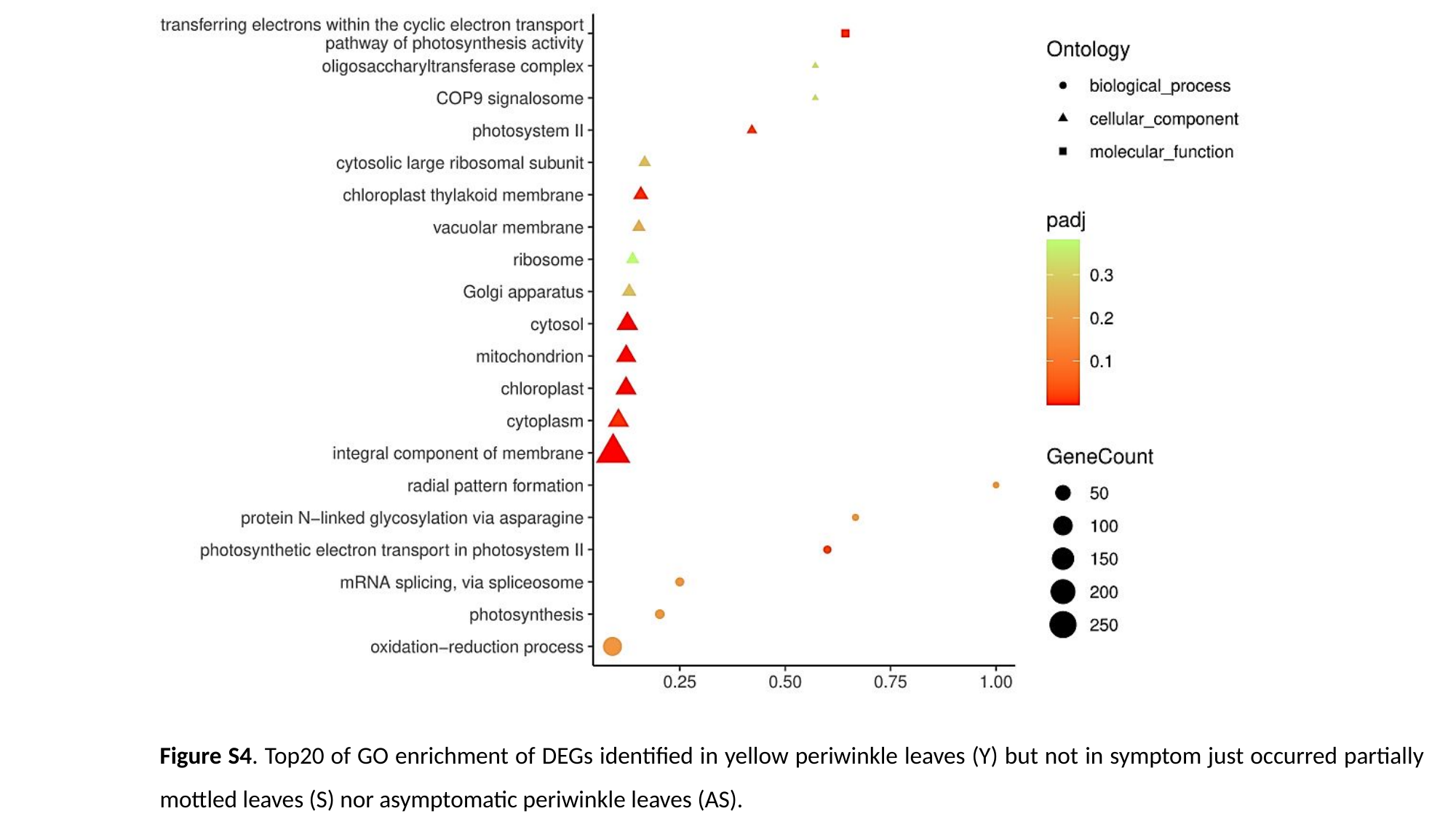

Figure S4. Top20 of GO enrichment of DEGs identified in yellow periwinkle leaves (Y) but not in symptom just occurred partially mottled leaves (S) nor asymptomatic periwinkle leaves (AS).

## Slide 5
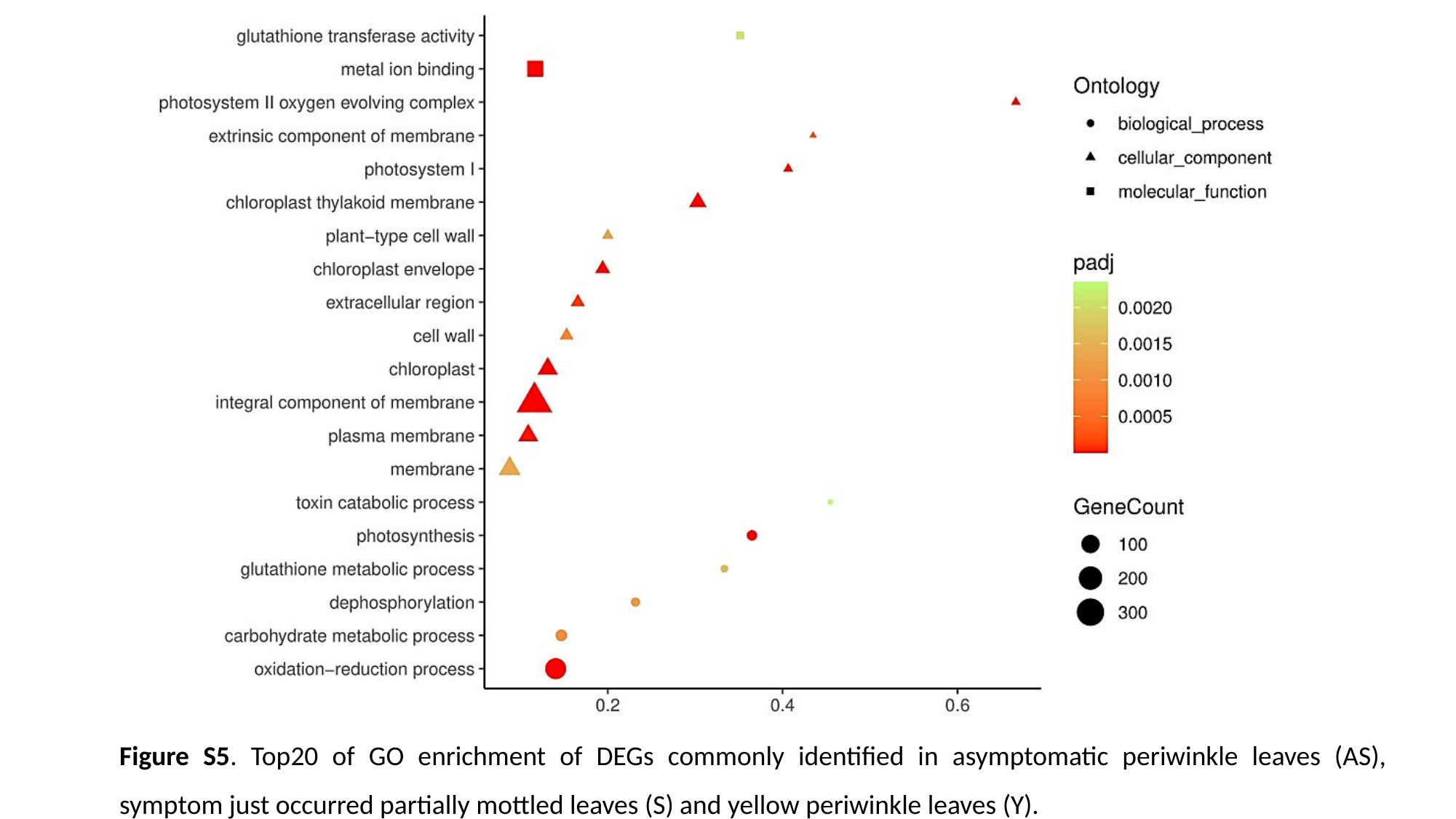

Figure S5. Top20 of GO enrichment of DEGs commonly identified in asymptomatic periwinkle leaves (AS), symptom just occurred partially mottled leaves (S) and yellow periwinkle leaves (Y).

## Slide 6
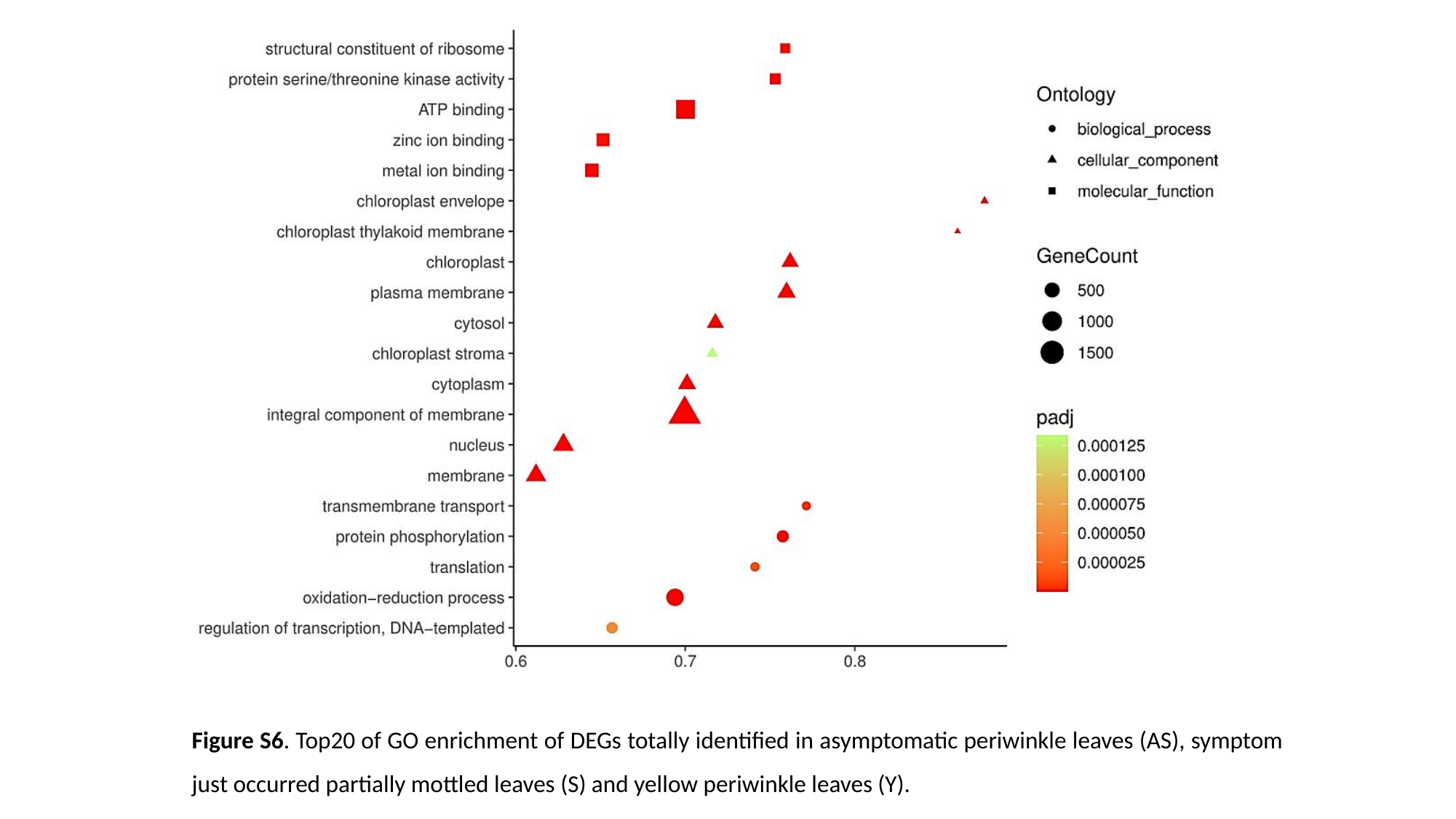

Figure S6. Top20 of GO enrichment of DEGs totally identified in asymptomatic periwinkle leaves (AS), symptom just occurred partially mottled leaves (S) and yellow periwinkle leaves (Y).

## Slide 7
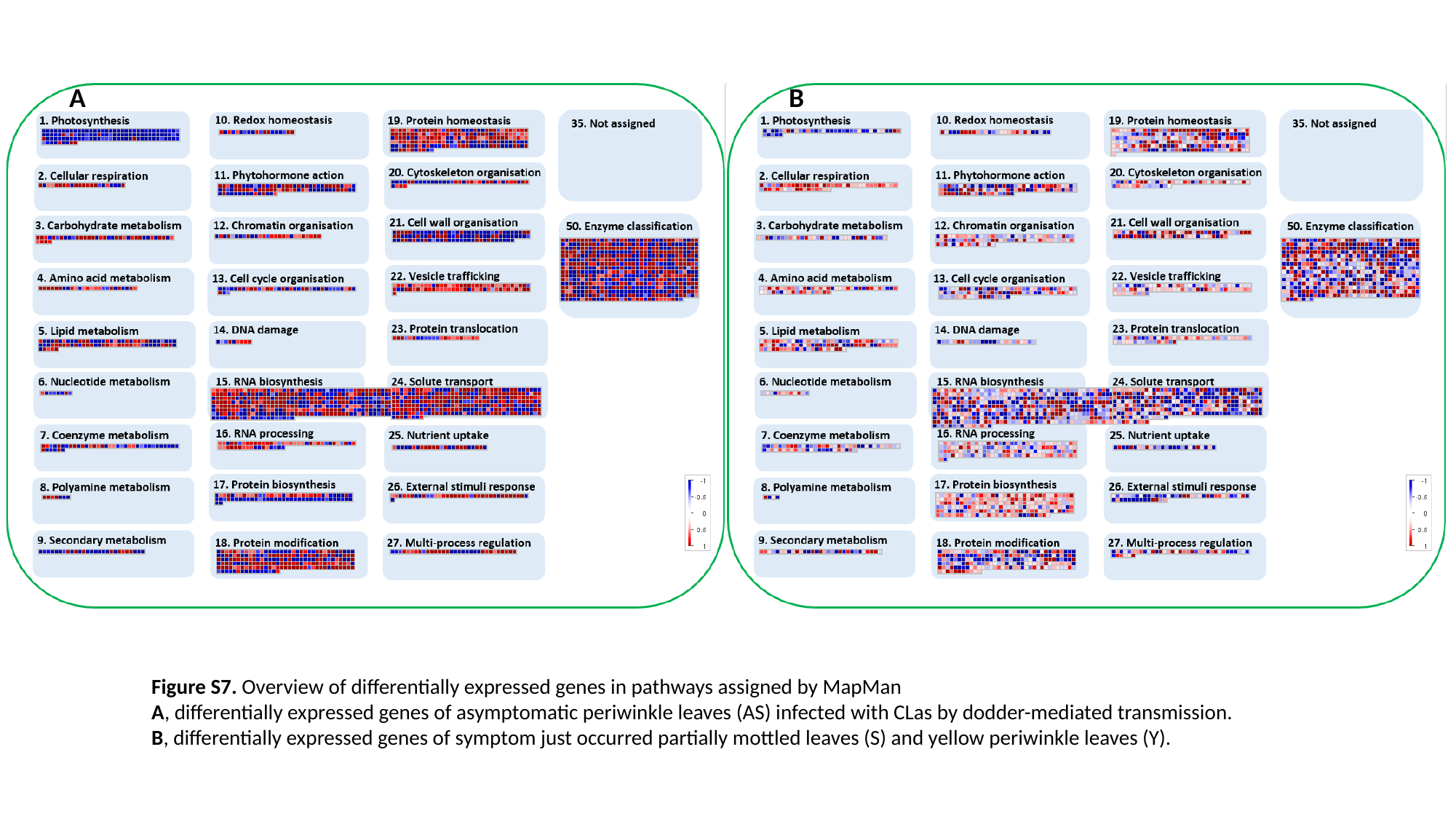

A
B
Figure S7. Overview of differentially expressed genes in pathways assigned by MapMan
A, differentially expressed genes of asymptomatic periwinkle leaves (AS) infected with CLas by dodder-mediated transmission.
B, differentially expressed genes of symptom just occurred partially mottled leaves (S) and yellow periwinkle leaves (Y).

## Slide 8
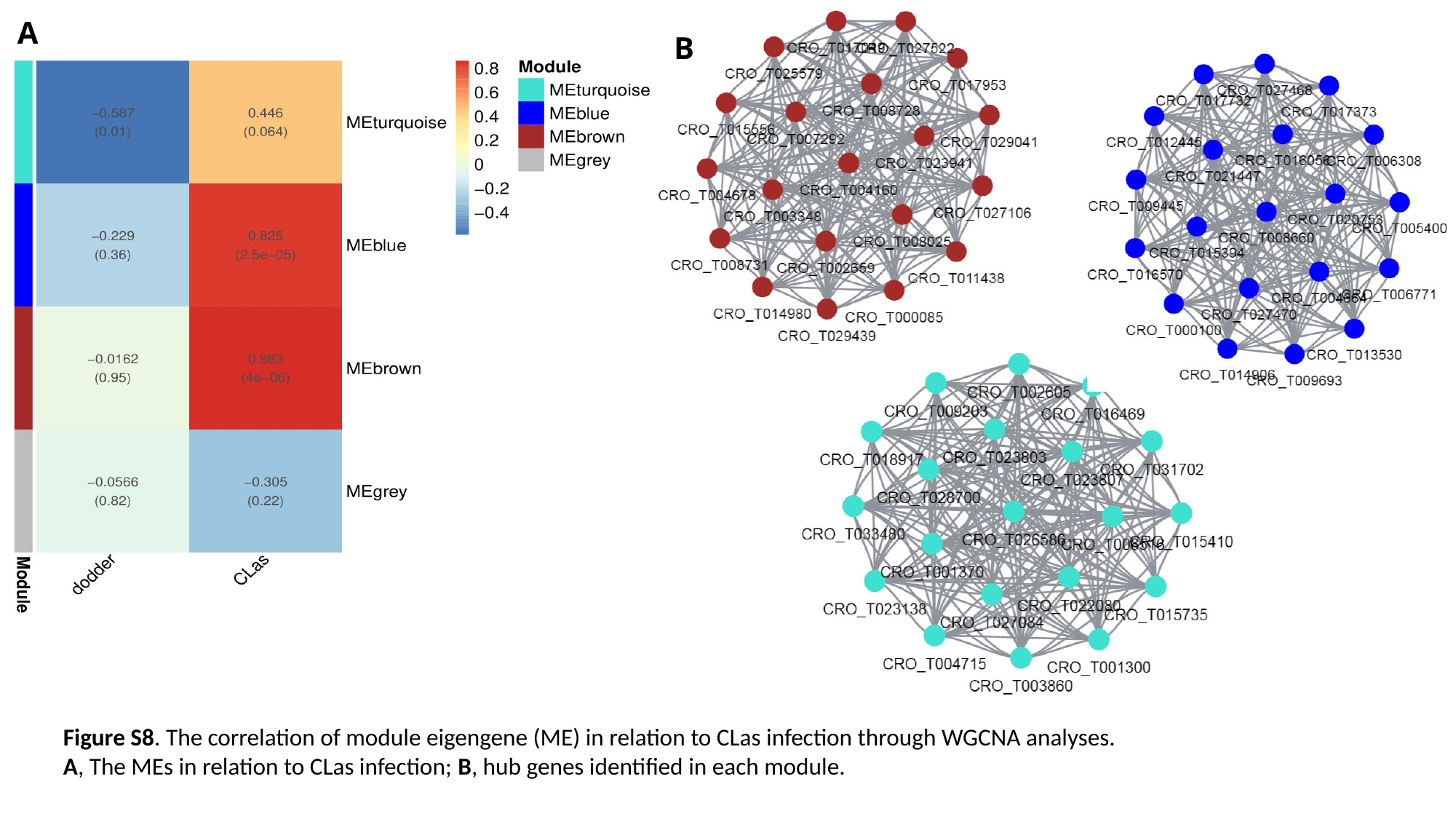

A
B
C
Figure S8. The correlation of module eigengene (ME) in relation to CLas infection through WGCNA analyses.
A, The MEs in relation to CLas infection; B, hub genes identified in each module.

## Slide 9
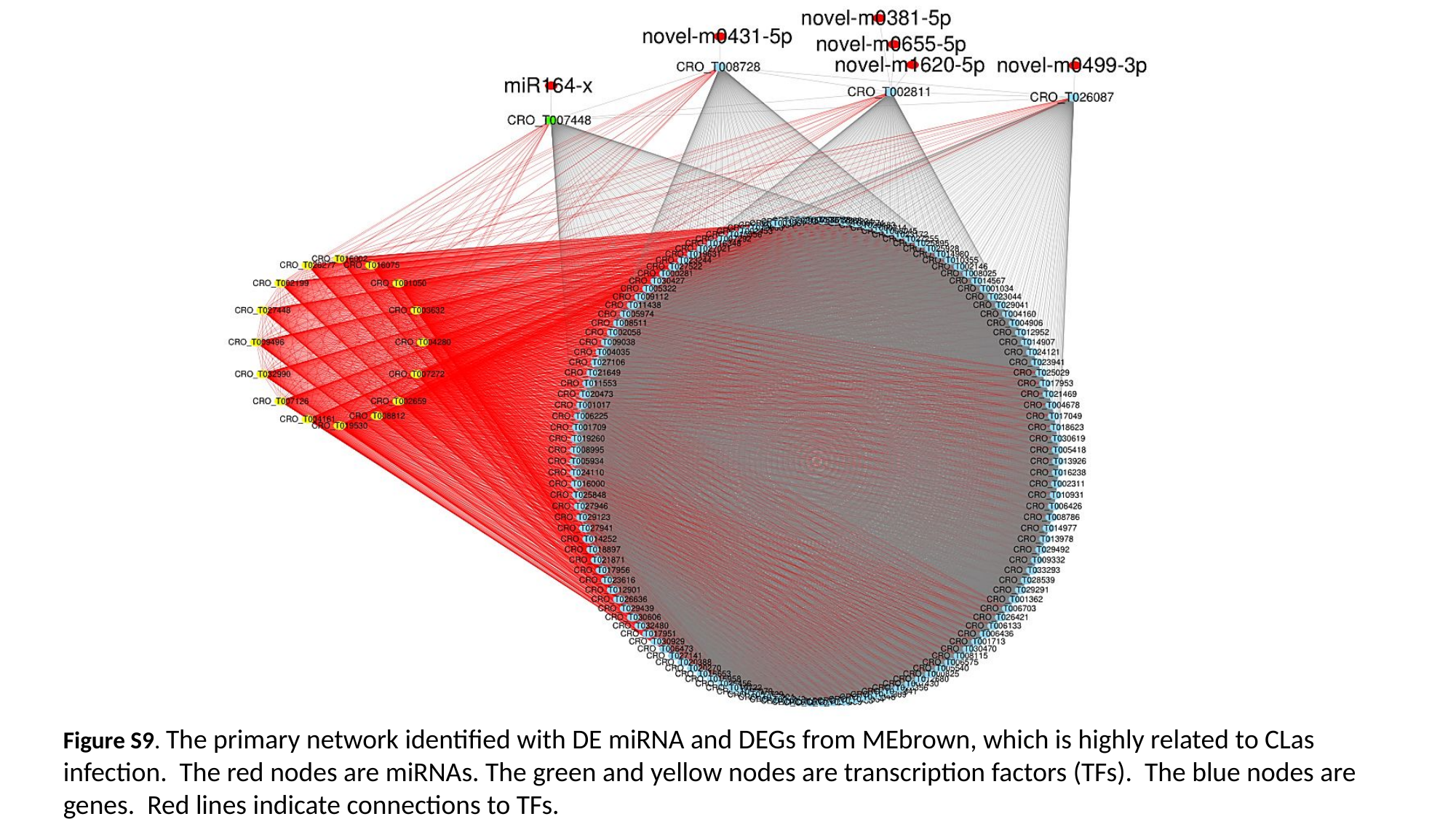

Figure S9. The primary network identified with DE miRNA and DEGs from MEbrown, which is highly related to CLas infection. The red nodes are miRNAs. The green and yellow nodes are transcription factors (TFs). The blue nodes are genes. Red lines indicate connections to TFs.

## Slide 10
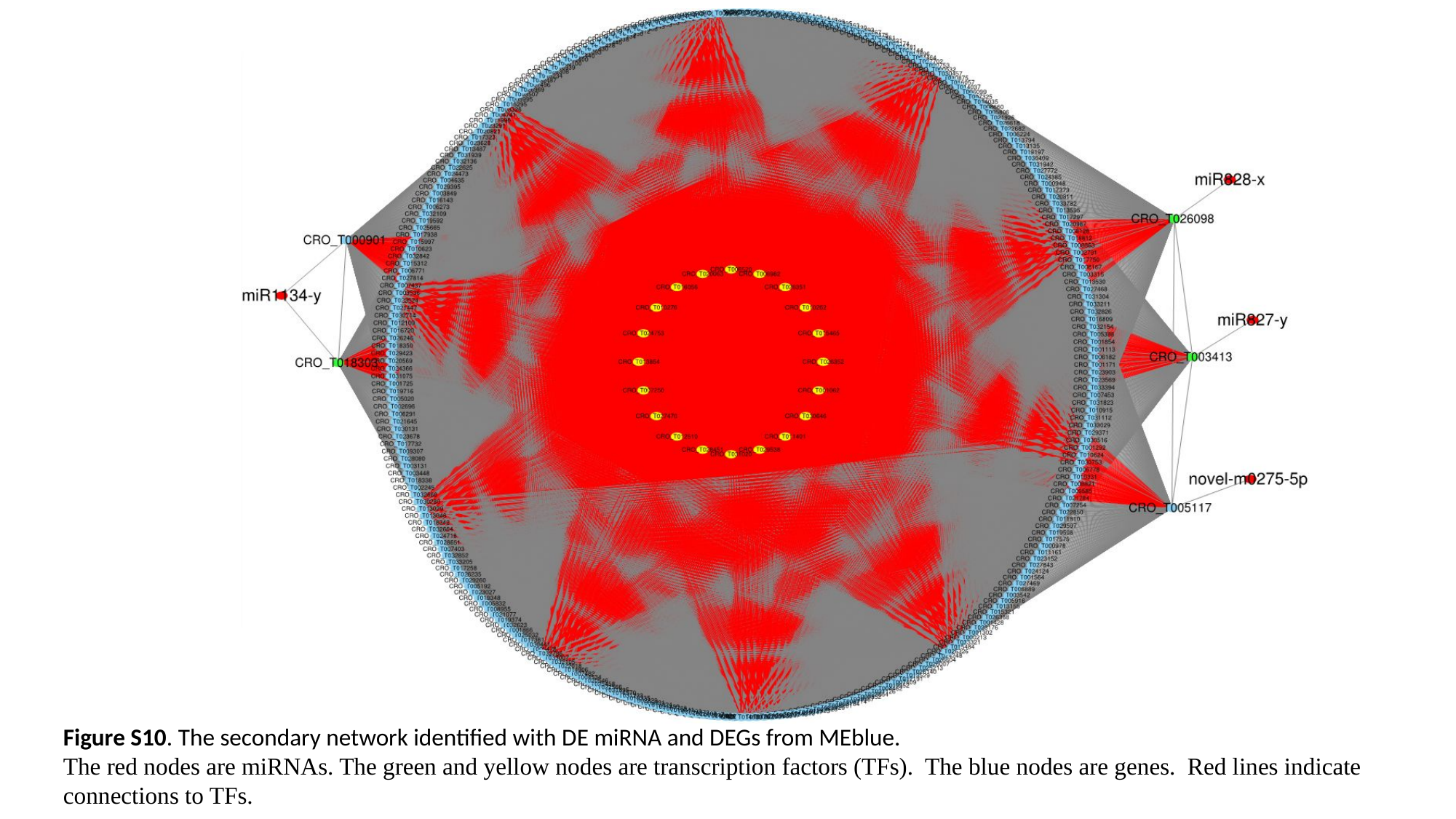

Figure S10. The secondary network identified with DE miRNA and DEGs from MEblue.
The red nodes are miRNAs. The green and yellow nodes are transcription factors (TFs). The blue nodes are genes. Red lines indicate connections to TFs.

## Slide 11
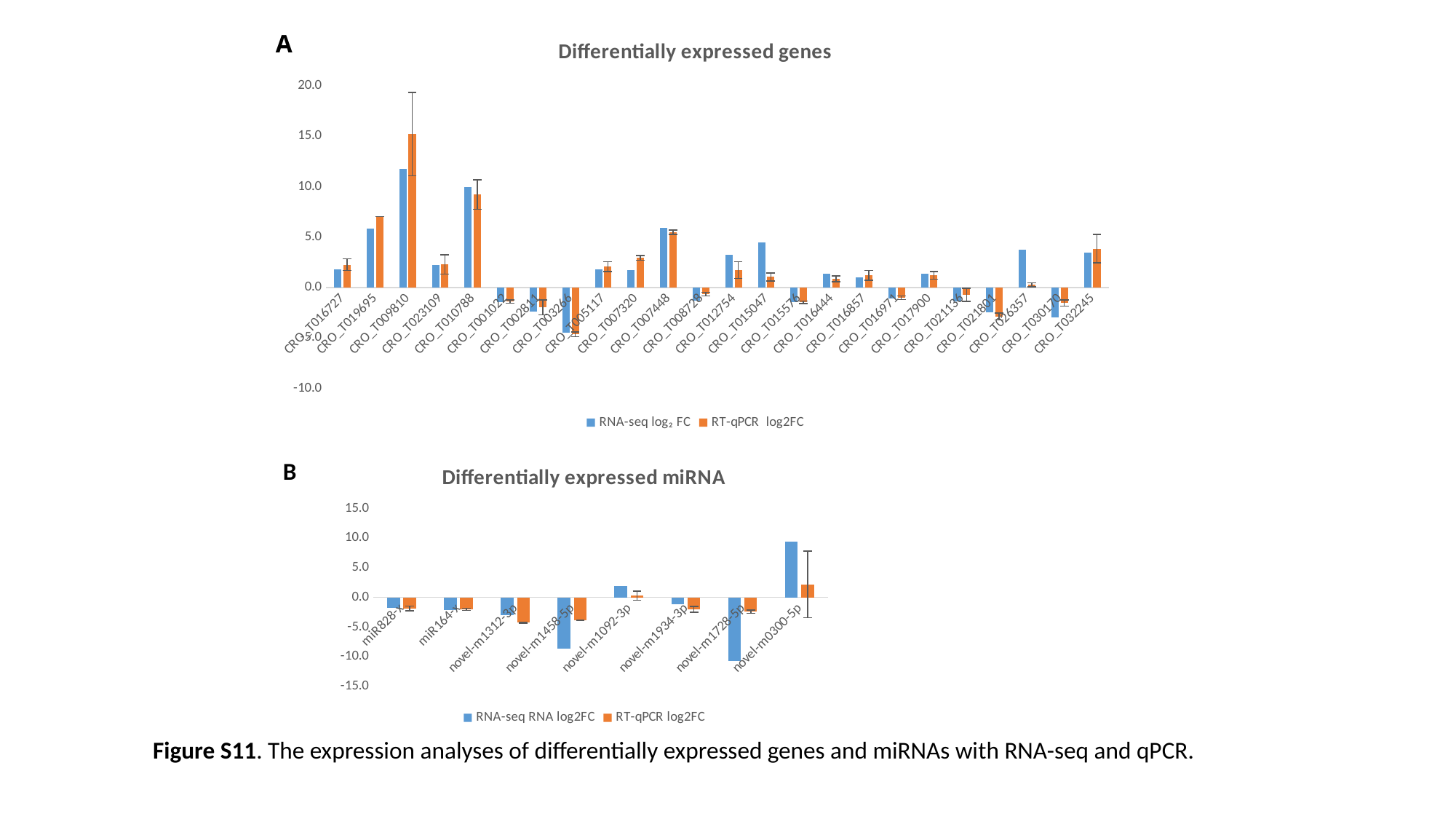

### Chart: Differentially expressed genes
| Category | RNA-seq log₂ FC | RT-qPCR log2FC |
|---|---|---|
| CRO_T016727 | 1.768336911223231 | 2.263202110708917 |
| CRO_T019695 | 5.842929887955229 | 7.02 |
| CRO_T009810 | 11.7399418828901 | 15.2 |
| CRO_T023109 | 2.24546625497001 | 2.28 |
| CRO_T010788 | 9.978454876422015 | 9.215089442474136 |
| CRO_T001022 | -1.441629505202088 | -1.3936633610043299 |
| CRO_T002811 | -2.39572410413902 | -1.9742480130504336 |
| CRO_T003266 | -4.4450539594586 | -4.64 |
| CRO_T005117 | 1.8084131810775048 | 2.0710570861928512 |
| CRO_T007320 | 1.6913980420407917 | 2.9365750389223413 |
| CRO_T007448 | 5.907566629993428 | 5.48 |
| CRO_T008728 | -1.2812916412592705 | -0.6613959798411447 |
| CRO_T012754 | 3.268815364501733 | 1.73 |
| CRO_T015047 | 4.474337373504385 | 1.04 |
| CRO_T015576 | -1.4564109301619277 | -1.46 |
| CRO_T016444 | 1.3808833187956489 | 0.86 |
| CRO_T016857 | 1.0378513221928733 | 1.2029793314894417 |
| CRO_T016971 | -1.0965917256908777 | -1.02 |
| CRO_T017900 | 1.3364050781961294 | 1.21 |
| CRO_T021136 | -1.3452121573313987 | -0.725675430644632 |
| CRO_T021801 | -2.474265562264261 | -2.866530341949551 |
| CRO_T026357 | 3.7142480875553585 | 0.26 |
| CRO_T030170 | -2.955594169352533 | -1.5522284014851704 |
| CRO_T032245 | 3.439008092062653 | 3.85 |B
### Chart: Differentially expressed miRNA
| Category | RNA-seq RNA log2FC | RT-qPCR log2FC |
|---|---|---|
| miR828-x | -1.80945080976065 | -1.83 |
| miR164-x | -2.13636524247038 | -2.01 |
| novel-m1312-3p | -2.94930353972988 | -4.242422199985089 |
| novel-m1458-5p | -8.5858138403052 | -3.852684921736776 |
| novel-m1092-3p | 1.97446043611736 | 0.3 |
| novel-m1934-3p | -1.20138491520822 | -1.99 |
| novel-m1728-5p | -10.671001392533 | -2.376290493283936 |
| novel-m0300-5p | 9.4690176674665 | 2.23 |Figure S11. The expression analyses of differentially expressed genes and miRNAs with RNA-seq and qPCR.

## Slide 12
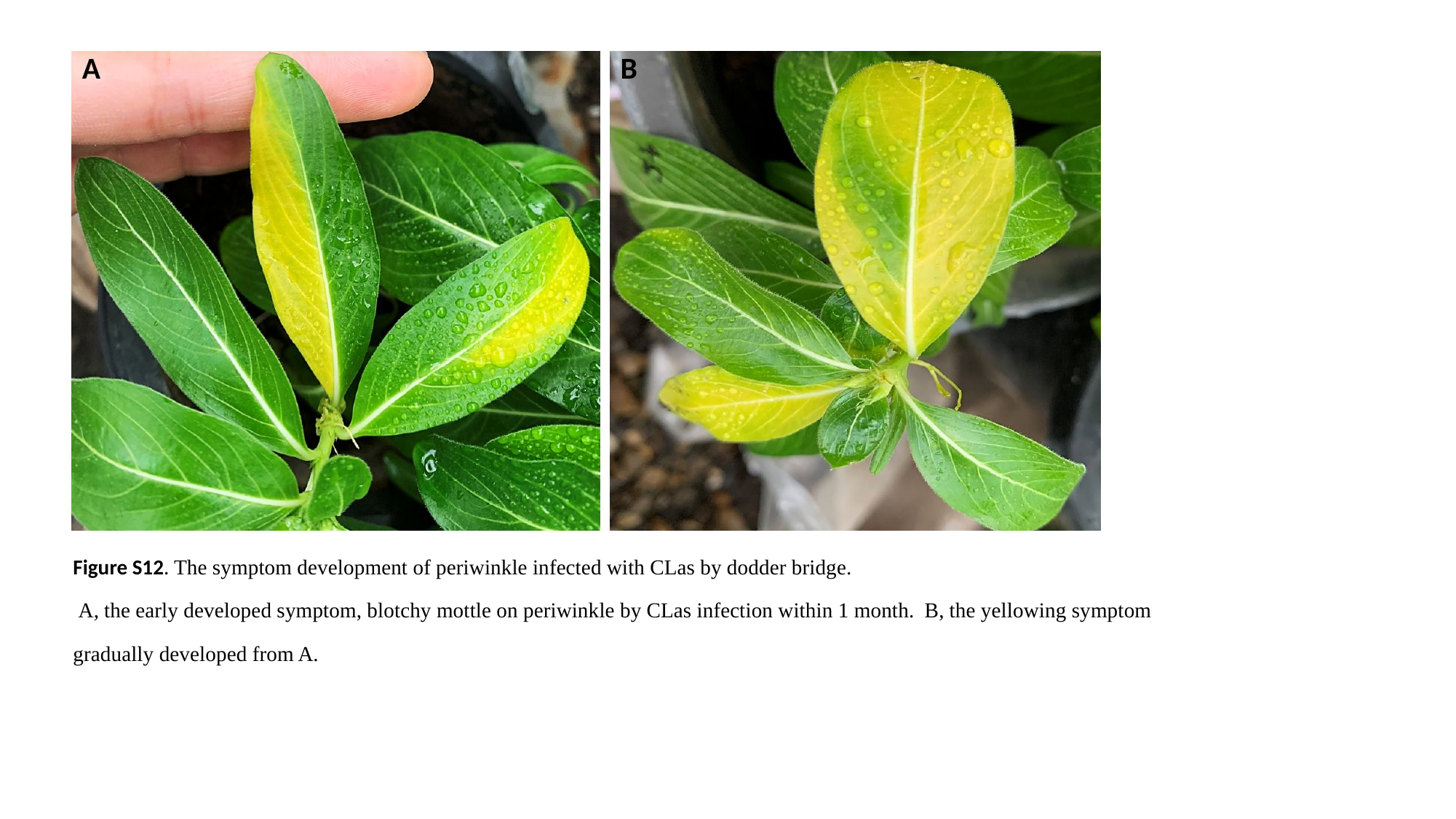

A
B
Figure S12. The symptom development of periwinkle infected with CLas by dodder bridge.
 A, the early developed symptom, blotchy mottle on periwinkle by CLas infection within 1 month. B, the yellowing symptom gradually developed from A.
